# Exploring peer tutoring experiences in improving nursing students’ academic success and performance: A qualitative study among Emirati undergraduate nursing students

**DOI:** 10.12688/f1000research.172023.1

**Published:** 2025-12-19

**Authors:** Jacqueline Maria Dias, Fatema Salim Bati Salim Al Kaabi, Sara Tamim Al Hooti, Nada Osamah Hammadi, Rana Karim, Nabeel AlYateem, Fatma Reefat Ahmed, Mini Sara Abraham, Vidya Seshan, Muhammad Arsyad Sub, Ambrose Richard Dias, Richard Mottershead

**Affiliations:** 1 Department of Nursing, College of Health Sciences, University of Sharjah, Sharjah, United Arab Emirates; 2 University of Wollongong in Dubai, Dubai, United Arab Emirates; 3 Behavioural Science Institute, SEHA, Sakina, Al Ain, United Arab Emirates; 4 College of Nursing, University of Baghdad, Baghdad, Iraq

**Keywords:** Peer tutoring, nursing student, undergraduate, nursing education, academic success, performance, qualitative, emotional resilience, United Arab Emirates, social learning theory

## Abstract

**Background:**

Peer tutoring, a pedagogical approach in which students assist one another in the learning process, has demonstrated strong educational and psychosocial benefits in health professions education. It enhances cognitive development, communication, self-efficacy, and social connectedness. Within nursing education, peer tutoring encourages reflective learning and professional growth. However, limited research has explored its impact within culturally and linguistically diverse student populations in the United Arab Emirates (UAE).

**Aims:**

This study explored the experiences of nursing student tutors and tutees at the University of Sharjah, evaluating their impact on academic development, emotional resilience, interpersonal connections, and the overall learning experience within a culturally and linguistically diverse population.

**Methods:**

This qualitative study used semi-structured interviews with a purposive sampling of 20 undergraduate nursing students (6tutors and12 tutees) at the University of Sharjah. Data were analyzed inductively through thematic content analysis following the COREQ guidelines.

**Results:**

Three main themes emerged: (1) Tutors’ perspectives and experiences, (2) Tutees’ perspectives and experiences, and (3) Barriers to peer tutoring. Peer tutoring was found to enhance academic comprehension, communication, and confidence, while also fostering collaboration and empathy. Challenges included language barriers, inconsistent commitment, and limited time for relationship building

**Conclusions:**

Peer tutoring represents an effective, student-centered learning strategy that supports academic success, promotes psychological safety, and nurtures professional competencies among nursing students. Findings underscore the need for structured, institutionally supported peer tutoring programs that address linguistic diversity, ensure tutor preparation, and integrate emotional and cultural sensitivity into nursing curricula.

## Introduction

Nursing education aims to equip students with the knowledge, skills, and professional attitudes necessary to deliver safe, competent, and compassionate patient care. Nursing is not just about knowledge, but also about effective communication, critical thinking, and problem-solving. Nursing students often find it challenging to enter a professional field like nursing due to its complex curriculum and high academic standards, especially during their first year ( Choi et al., 2021). According to Cupelli & Colalillo (2025), peer-led activities significantly enhance nursing students’ ability to evaluate clinical scenarios, provide constructive feedback, and develop essential teamwork skills. A safe, structured, and supportive peer learning environment helps students perceive feedback as a tool for growth, rather than criticism, thereby enhancing their learning experience ( Mu’alifah, Pribadi & Elliya, 2023). Peer tutoring teaches students to value each other’s efforts, learn from shared challenges, and celebrate growth together ( Astuti, Sapriya, & Jupri, 2024). Peer tutoring has become an integral part of nursing education, aiming to improve students’ critical thinking, knowledge retention, and confidence ( Al Yahyaei et al., 2024; Cupelli & Colalillo, 2025; Herinek, Woodward-Kron, & Ewers, 2024; Kang, Lee, & Joung, 2021). It is also referred to by various terms, including peer teaching, peer instruction, and peer mentoring, which enable students to support one another’s understanding, ideas, and skills ( Choi et al., 2021).

Peer tutoring, based on social learning theory, is a teaching method that aims to improve knowledge and understanding through student interactions ( Palsson et al., 2017; Taffurelli et al., 2025). Peer tutoring involves a structured role rotation between tutors and students, where individuals from the same or different academic years take part ( Gazula et al., 2017). The benefits of students acting as peer tutors are well known, including improvements in metacognition, increased student responsibility, and the development of professional skills ( Burgess et al., 2016). It is designed to deepen and broaden students’ understanding of a subject through interactive small-group activities ( Curtis et al., 2016; Iwata & Furmedge, 2016). Peer tutoring programs are personalized learning methods that apply the principles of student cooperation, shifting away from lecture-centered, lecture-style teaching ( Kim et al., 2012). This educational approach specifically encourages mutual cooperation by engaging students in various interactive learning activities with their peers. This method helps improve academic achievement and supports student adjustment ( Colver & Fry, 2015). Consequently, these activities can effectively promote academic progress and assist nursing students in completing their studies ( Guerra-Martín et al., 2017). Previous research has demonstrated that peer tutoring yields several positive outcomes, including enhanced academic performance through improved learning skills, intellectual growth, supportive relationships, and personal development ( Li et al., 2018).

In nursing education, peer tutoring sessions can prepare students for real-world challenges and demonstrate that peer tutoring is not only beneficial to tutors but also helps tutors learn how to give constructive feedback and accept criticism. This practice promotes critical thinking and respect for the ideas of others—an important trait for nurses. For many nursing students, learning can feel overwhelming. Peer tutoring provides them with a supportive and interactive way of learning ( Wierzbinski-Cross, 2017). Kim et al. (2021) proposed that peer tutoring provides nursing students with an opportunity to ask questions, resolve conflicting information, and enhance their confidence in their knowledge and skills. Peer tutoring is a simple yet powerful way for students to support one another’s growth, learning, and skill development, ultimately increasing their readiness. In nursing, where teamwork and responsibility are essential, peer tutoring is an effective method for implementing these values while enhancing academic performance ( Kang et al., 2021). It involves students reviewing and providing feedback on each other’s work. Traditional teaching methods, although essential, may not fully meet the diverse learning needs of all students, prompting calls for innovative, evidence-based educational strategies ( Al Yahyaei et al., 2024; Ephraim, 2020; Hampe et al., 2024). However, some challenges still exist. Many students report that peer tutoring fosters a sense of belonging and decreases feelings of isolation ( Kim et al., 2021), while others have expressed discomfort with peer evaluation, raising concerns about potential shaming or judgment ( Kang et al., 2021). This highlights the need for a safe, well-structured, and supportive environment that fosters peer learning, where feedback is viewed as an opportunity for growth rather than criticism ( Muthalib et al., 2023) and Mottershead & Alonaizi (2022). Moreover, peer tutoring helps develop professional values such as empathy, respect for diverse perspectives, and collaborative problem-solving skills, which are vital in nursing practice ( Cupelli & Colalillo, 2025; Koo, An, & Lee, 2024).

While peer tutoring offers benefits for both general and nursing students, potential challenges should be recognized ( Palsson et al., 2017). To maximize effectiveness, creating a well-defined organizational structure for sessions is vital. This framework should promote a sense of purpose and confidence in students, while also boosting their knowledge and self-efficacy. Psychological empowerment plays a crucial role in this context, as it enables students to feel purposeful, capable, and influential throughout their educational journey ( Bradbury-Jones, Irvine & Sambrook, 2010).

### Study purpose

Despite its well-known benefits, the impact of peer tutoring on learning outcomes, student confidence, and social integration within the UOS nursing education framework has not been thoroughly examined. Studies have demonstrated that peer tutoring is an effective method for decreasing the failure rate of nursing students’ courses and enhancing their academic success. It serves as an additional tool to address challenges associated with online-based education ( Kim et al., 2021). This study explored the experiences of nursing student tutors and tutees at the University of Sharjah, assessing their impact on academic development, emotional resilience, interpersonal connections, and the overall learning experience within a culturally and linguistically diverse population of nursing students. Understanding the impact of peer tutoring on students’ academic experiences and professional readiness is crucial for nursing educators and administrators seeking to enhance teaching methods and student outcomes. The results of this study can help refine educational practices at the University of Sharjah and support the future development of academically skilled, reflective, and collaborative nursing professionals.

## Methods

### Design

This study employed a descriptive qualitative design to gain a deeper understanding of how undergraduate nursing students at the University of Sharjah (UoS) experience peer tutoring. Qualitative research explores individuals’ interpretations and meanings of social phenomena within specific contexts ( Grossoehme, 2014). This study employed qualitative content analysis methods, allowing for a detailed examination of students’ lived experiences. Content analysis, as an interpretive process, examines the topic and context, identifying similarities and differences within and across various texts ( Creswell & Poth, 2018). By adopting a qualitative research design, researchers can gain a deeper understanding of the complex and comprehensive nature of the studied topic ( Creswell, 2012) and Mottershead et al. (2024). Additionally, the researchers followed the COREQ qualitative study guidelines ( Tong et al., 2007).

### Setting and participants

This study was conducted between January and March 2025 at the College of Health Sciences, University of Sharjah, in the United Arab Emirates. The study included 17 undergraduate nursing students, comprising both tutors and tutees (6 tutors and 11 tutees), who participated by sharing their personal thoughts and stories. The inclusion criteria for the study were 11 tutees with a GPA below 2.5, and it included 6 tutors with a GPA above 3.5.

### Research instrument

The research instrument comprised two components: a self-designed questionnaire to gather demographic information and a semi-structured interview. The questions were developed through an extensive literature review. After formulation, they were validated by faculty members, including Dr. JMD, who has substantial tutoring experience. The literature review employed MeSH terms such as peer tutoring, emotional resilience, language barrier, and study methods. Following question development, a pilot test was conducted with 5 tutors and 11 tutees. Their feedback helped refine the questions, and the study ultimately addressed the research questions specified.

### Questions for the tutors

1. Describe your experience of peer tutoring.
2. Were you able to clarify the students’ needs and concerns?
3. What strategies did you use to help the students?
4. How beneficial was this experience for you?
5. Would you repeat this experience and why?
6. Do you feel that the students accepted your feedback, and why?
7. How did tutoring impact your patience level as well as your ability to work with different learning styles?
8. Did you build any connections with the students you taught?
9. Did you share your experience with them?
10. Do you think it will be better to get paid for your service?

### Questions for the tutees

1. Describe your experience of receiving peer tutoring.
2. Were you nervous receiving peer tutoring?
3. Did you benefit from it academically and socially?
4. How many hours do you spend in lectures on top of that?
5. How many hours do you spend in university studying?
6. Do you prefer studying individually or with a group?
7. When were you aware that you needed peer tutoring, and would you continue with it?
8. Did you benefit from the experience, and in what way?
9. Did you respect the time the peer tutor gave you?
10. Would you consider becoming a peer tutor yourself?
11. Would you review and provide feedback on your own work as well as the work of other students?

### Data collection

Semi-structured interviews with students and personal tutors, lasting 45 to 60 minutes, were conducted remotely through Microsoft Teams. With permission, these interviews were video recorded and transcribed exactly as spoken. The interview guides for both groups were piloted earlier, but no changes were made after the pilot. To gain deeper insight into the genuine experiences of nursing students engaged in peer tutoring at the University of Sharjah, we used a qualitative approach. We employed two primary techniques to collect the required data: open-ended surveys and semi-structured interviews. We conducted semi-structured interviews with tutees and peer tutors. In addition to providing a framework, this format allows students to discuss their experiences freely and organically. We inquired about the methods they employed, the difficulties they encountered, and the effects peer tutoring had on their social lives, confidence, and academic achievement. By allowing students to express themselves in their own words, we were able to get deep, significant insights that a typical questionnaire might have overlooked. We developed a Google Form survey to gather demographic data and open-ended answers in addition to the interviews. Participants were given the chance to write about their experiences and reflect on them through the survey. Additionally, it enabled us to connect with students who might have felt more at ease expressing their opinions anonymously as opposed to in person. Student participants were assured that their information would be kept confidential and provided with informed consent prior to participation.

### Data analysis

This study employed inductive content analysis ( Elo & Kyngäs, 2008) to analyze the data. In the preparation phase, a single word or phrase was selected as the analysis unit, following recommended procedures. The detailed analysis during the organizing stage consisted of several steps: First, the transcribed interview data were read repeatedly to grasp their content and overall flow; common words or phrases were identified and coded. Second, these systematically identified codes were reviewed and grouped based on shared features. Third, the categorized content was analyzed iteratively, with labels assigned to capture their meanings and relevance. Finally, broader abstract categories were created to organize descriptions related to the research topic. Additionally, we reviewed and clarified the meanings of concepts associated with the topics from the table assessment to ensure data validity and appropriateness.

### Ethical consideration

The University of Sharjah Research Ethics Committee (REC) approved this study (Number: REC-25-02-10-02-S). Before each interview, the process was explained to participants, and written informed consent was obtained. Participants were informed that their participation was voluntary and could be withdrawn at any time; if they chose to withdraw, their data would be removed from the study. No participant chose to withdraw. Interviews were conducted online to ensure participants felt comfortable in a quiet, private setting, encouraging open sharing of opinions. Participants were informed they would not receive compensation and that their participation would be kept anonymous and confidential. Confidentiality involves keeping participants’ information private, with restrictions on the sharing of data. They were assured that their data would not be duplicated or shared without permission. Personal identifiers were removed to ensure anonymity; access to recordings and transcripts was limited to researchers. All sensitive data were securely stored in the principal researcher’s office at the University of Sharjah. The data will be retained for five years after the study concludes, after which all materials will be destroyed, transcripts will be shredded, and recordings will be demagnetized.

## Results

Qualitative content analysis of the interviews uncovered both advantages and disadvantages of the peer tutoring process. Students highlighted the benefits of promoting deep learning, cooperation, and personal growth, while also noting some challenges encountered during peer-tutoring activities. The majority of tutor participants were between 21 and 23 years old, and tutees were between 18 and 20 years old. All interviewees stated that they felt comfortable providing feedback to the tutees. Most notably, all participants expressed a willingness to tutor again next semester, and the majority agreed to be interviewed again in the future. Qualitative content analysis identified three main themes: (1) Tutors’ perspectives and experiences, (2) Tutees’ perspectives and experiences, (3) Peer tutoring barriers. Each main theme has subthemes. Tutors reported five themes, and tutees identified three themes. Peer tutoring barriers have four subthemes. All themes were found to be closely related and appeared to have a direct influence on peer relations. Peer tutoring provides valuable opportunities for developing skills essential for future professional practice, enhancing self-efficacy, promoting psychological safety, and fostering collaboration among nursing students.

### Theme 1: Tutors’ perspectives and experiences

***Subtheme: Positive experience of teaching and learning***

Overall, the enjoyment of teaching and learning emerged as a central and motivating force behind the peer tutors’ engagement. Some tutor participants described their role not only as a chance to support others but also as something personally fulfilling and enjoyable. For some, the joy of teaching began well before university, and this peer tutoring opportunity provided them with a chance to revisit and deepen that interest. As one participant shared:

*Actually, my experience with the program was really enjoyable*… *I really enjoy teaching. I have always enjoyed explaining things to others since high school*… *Honestly, I aspire to continue my studies after graduation, pursuing a master’s and PhD, and then become a university professor, as I truly love teaching. (Tutor 1)*

***Subtheme: Satisfaction from helping others***

Beyond academic interests, participants also noted the intrinsic satisfaction that came from helping others grasp difficult content and witnessing their peers’ progress. Peer tutoring was perceived not only as a method of transferring knowledge, but as a mutual process of growth and discovery:

*It was a good experience… I enjoy helping others understand things better and sharing my knowledge… Yes, I would definitely do it again because I enjoyed helping someone and sharing tips… It also helped me grow and improve my skills. (Tutor 2)*

The sense of fulfillment that came from supporting peers contributed to a deeper motivation to continue in similar roles. For some, this joy was rooted in a broader love of learning and a desire to contribute to the success of others:

*It was an amazing experience… It’s something that I like. I enjoy the teaching from my school days, and I continue to appreciate it now in university, particularly in a major that I like, with subjects that I enjoy. So, I like to share the knowledge I have. (Tutor 3)*

***Subtheme: Collaborative learning environment***

Participants also reported that the experience created a positive and collaborative learning environment, where mutual support lessened academic pressure and created a sense of community:

*Definitely yeah… As I said, I used to enjoy it and it felt great to see my classmates become more confident… it made studying less stressful because we were all in it together. (Tutor 4)*

In addition to deep learning competencies, participation in the peer-tutoring program enhanced students’ collaborative learning skills. Both tutors and tutees recognized that it was a collaborative process.

*…I believed I comprehended the material; nevertheless, when the tutee or tutor posed additional inquiries, I recognized my lack of understanding. This enabled me to recognize my own deficiencies. We collaboratively sought information from diverse sources, and we both acquired knowledge from it. (Tutor 6)*

***Subtheme: Communication skills***

For some tutors, the enjoyment was also personal and emotional, especially when tutoring close friends or classmates they cared about:

*… Yeah, I enjoy… I’ll keep doing it because I love it… It provides me with opportunities for interpersonal relationships. Ever since I was a kid, I’ve loved learning. I enjoy helping others understand things better and sharing my knowledge. (Tutor 2)*

Also, this was a frequently mentioned area by participants. Students noted that they improved respect and communication within the tutor–tutee relationship. Here are some of their comments.

*…. I developed a range of communication skills. In the past, I would avoid difficult patients, but through peer-tutoring, I learned to confront challenges and focus on effective communication with tutees. Now, when I encounter uncooperative patients, I try to engage with them… (Tutors 4)*

Students also indicated that they had developed greater openness, understanding, and supportiveness in their didactic relationships.

*Yes, it is. I’ve learned that practicing patience is important, especially when teaching health lessons to our clients. Additionally, I’ve realized the value of being a good listener and should pay attention when others want to express themselves. (Tutor 2)*

***Subtheme: Develop problem-solving and critical thinking skills***

Tutors valued the chance to ask questions or respond to inquiries that encouraged their critical thinking during sessions. Interviews clearly showed that the tutoring process helped develop problem-solving and critical thinking skills.

*… My tutee and I talked about why we follow certain methods. I used to follow the teacher’s or literature’s instructions exactly, but now I wonder if that’s needed. After being asked about this… I began to critically assess my actions and consider other options. (Tutor 4)*

***Subtheme: Building relationships***

An important aspect of the peer tutoring experience, as described by participants, was the development of relationships between tutors and the students they supported. Many tutors noted that the interactions extended beyond academic instruction and contributed to a sense of connection, trust, and mutual support. For some, the relationship evolved naturally into a friendlier dynamic, breaking the traditional boundaries between tutor and student. This shift fostered a more relaxed and open learning environment:

*Of course, we became more like friends rather than just a tutor-student relationship… you know, she was open to my advice, asked questions, and seemed grateful for the help. (Tutor 2)*

Some tutors found that, in building rapport, students felt comfortable enough to ask about a variety of topics—even those outside the formal tutoring sessions or the nursing curriculum. This showed the trust students placed in their tutors and highlighted the informal mentorship aspect of the experience:

*Some of them, for example, she can sometimes ask for advice on other subjects in different majors. She asked me about clinicals —how many hours, and how do you manage? I explained it to her. At first, you may struggle, but you can adapt. Yes, and because we will have future classes. I told her I would start consolidation, and I’ll keep her updated. (Tutor 5)*

In some cases, pre-existing friendships enhanced the comfort and effectiveness of the tutoring session. However, tutors also acknowledged that online sessions and timing constraints (e.g., during exams) could limit opportunities for deeper interaction:

*Of the students that I taught, some were my friends, so I had a relationship with them already… but others, no, because the session was online and during exam period. (Tutor 3)*

### Theme 2: Tutee’s perspectives and experiences

***Subtheme 1: Positive academic impact***

In exploring the experiences of students who received peer tutoring, the first major theme identified was the positive academic impact. Many participants noted that peer tutoring significantly enhanced their understanding of complex subjects, improved their performance in exams, and provided new study strategies that supported their academic success. Several students shared that tutoring sessions simplified confusing topics and helped them master challenging coursework. For example, one participant mentioned:

*Some calculations in biostatistics were confusing and overlapped, but the peer tutor was able to simplify the differences, and I understood them clearly. (Tutee 9)*

Similarly, another student highlighted the academic improvements:

*Yeah, I did benefit from the experience as I got in my midterm 25/30, which was good for me, and I learned how to change my studying style from only reading to watching videos and understanding, not just memorizing. (Tutee 2)*

Another tutee emphasized the lasting academic effect, stating:

*…Yeah, as I mentioned earlier, my GPA got higher and I’m a transfer student, so it really helped me*… *(Tutee 4)*

***Subtheme 2: Hesitancy and emotional issues***

Another prominent theme that emerged was the initial nervousness students felt before receiving peer tutoring. Several participants disclosed that they were anxious at first due to fears of judgment, the stigma of seeking help, or simply the uncertainty of working with someone new.

*…Being honest, I was nervous about the stigma of how a smart student was receiving peer tutoring. (Tutee 1)*

Other participants shared that the nervousness stemmed from unfamiliarity with their peer tutors, as described by one tutee:

*At first, yes, because I didn’t know the student well. But as the session went on, I got used to it. Yeah… but I think it was because it was my first time. (Tutee 10)*

Despite these initial fears, many noted that their anxieties were quickly alleviated after the first session. The friendliness and supportiveness of the peer tutors played a key role in easing their concerns, as illustrated by another student’s comment:

*Yes, I was a little nervous at the beginning because I didn’t know what to expect, and I was worried that I might not keep up. However, after the first session, I felt much more comfortable… Yes, at the beginning I was, but then after that… I was too confident with it. (Tutee 6)*

***Subtheme 3: Recognizing the need for support***

Tutee participants often became aware of their need for help when they struggled academically, faced upcoming exams, or felt lost after missing key lectures.

*… When I saw that my other colleagues were benefiting from her tutoring, and before my midterms, I was struggling in the course, I understood I needed help. When I realized that I needed help with my courses, my friend told me about it. I knew immediately that I needed it when I received it*… *(Tutee 4)*

Recognizing the importance of seeking help, many students reported that peer tutoring filled critical gaps in their learning and enabled them to regain control over their academic performance. Additionally, recognizing the need for support was a crucial step for students, enabling them to access timely assistance and improve their academic outcomes.

*… When I found myself lost and struggling to understand the lessons, that’s when I needed the tutoring… I was overwhelmed; I realized that I had trouble studying, and my grades were dropping significantly*… *When I didn’t understand some of the courses, as I took microbiology with H [a tutor], she helped me a lot. (Tutee 2)*

### Theme 3: Challenges in peer tutoring

Although students appreciated the tutoring process and recognized its utility, they also encountered frustrations and disappointments. The causes of these disappointments typically arise from a lack of engagement in learning, challenges with punctuality, insufficient dedication, inadequate knowledge, and discrepancies in expectations. While many tutors expressed positive experiences with teaching, several also reflected on the challenges they faced during the tutoring process. These challenges varied from academic and pedagogical barriers to communication issues and time constraints, highlighting the complexity of the tutoring role.

***Subtheme: Language barrier***

One of the most frequently noted difficulties was the language barrier—not in spoken language, but in the way course material was written and understood by students. Tutors observed that the primary struggle for many of their peers was not the core content itself, but deciphering academic language, especially in subjects like nursing.

*Through this experience, I also realized that most of the students struggle more with understanding the language of the subject rather than the subject itself. Honestly, patience is a quality that anyone who loves teaching or wants to be an educator must have, because we have multiple students with different levels of understanding and different learning styles. (Tutor 1)*

Other tutors spoke about logistical and relational barriers that hindered effective communication and rapport-building with the tutees. A lack of time and the limitations of online sessions—especially during stressful periods like exam season—were commonly cited.

*… There wasn’t enough time to build a relationship… the session was online and during the exam period. (Tutor 3)*

In contrast, tutors from English-speaking backgrounds recognized the language barrier but approached it in a different way. They focused on simplifying English explanations rather than incorporating Arabic, which sometimes limited the effectiveness of communication with certain tutees.

*… Sometimes I feel that the student is shy about saying they didn’t understand the English. I try to simplify, but it’s hard when they don’t speak up. (Tutor 2)*

***Subtheme: Passive learning***

Overall, the tutoring experience illuminated both the rewarding and demanding aspects of peer education. Tutors developed a deeper understanding of the diversity in student needs and the skills required to support meaningful learning, such as patience, clarity, and flexibility. Tutors found it exasperating when tutees arrived unprepared for sessions, yet anticipated reiterations of explanations already provided in lectures.

*During several meetings, the tutee/tutor failed to adequately prepare, necessitating a review of notes and explanations like to those provided by the instructor in class. This was distressing for me. (Tutor 3)*

Some tutors also faced frustration with students’ study habits, particularly when learners relied solely on passive techniques such as reading slides without deeper engagement.

*The student I taught was only reading through the slides*… *which in my opinion is useless… especially with anatomy…She was a little bit doubting… if you just read through the slides… You won’t recall much for the midterm. (Tutor 5)*

***Subtheme: Lack of punctuality and commitment***

Several participants indicated that coordinating a mutually convenient time was challenging due to their other commitments. Some tutors perceived the preparation of tutoring sessions as arduous. Punctuality was an issue for participants in peer tutoring.

*On some occasions, she was behind schedule, necessitating a 45-minute wait on my part. I was unable to locate her. It was challenging and distressing. I questioned whether there was an issue with me. Moreover, as the semester concluded, we were preoccupied with our tasks, complicating the scheduling of an appropriate time. I found the preparation for tutoring to be somewhat arduous. (Tutor 6)*

## Discussion

This study aimed to explore nursing students’ experiences and views on peer tutoring in the Bachelor of Science in Nursing (BSN) program at the University of Sharjah.

Through qualitative data gathered from semi-structured interviews and open-ended surveys, the study illuminated the multifaceted impact of peer tutoring on students’ academic performance, emotional well-being, social engagement, and professional development. The findings confirm the growing body of literature that recognizes peer tutoring not merely as a supplemental educational tool, but as an essential pedagogical approach that supports holistic student growth in nursing education ( Cupelli & Colalillo, 2025; Lockspeiser et al., 2008). The Bachelor of Science in Nursing (BSN) program at the University of Sharjah (UOS) is a comprehensive 137-credit-hour curriculum that combines theoretical knowledge with practical clinical experience. As part of ongoing efforts to improve academic readiness, UOS has increasingly adopted peer tutoring as a method to support student learning and academic success ( Subu et al., 2023). At the UOS, peer tutoring involves organized sessions where nursing students work together to review each other’s work, provide constructive feedback, and support each other’s academic growth. Research indicates that this approach not only enhances academic performance but also fosters key professional skills, including communication, teamwork, and reflection ( Cupelli & Colalillo, 2025; Kang et al., 2021; Wierzbinski-Cross, 2017).

The social dynamic between tutors and tutees also played a key role in shaping the effectiveness of peer tutoring. As students worked together, the majority reported that their relationships evolved beyond formal academic support into genuine friendships or mentorships. This shift fostered a more relaxed and open environment where students felt comfortable asking questions, sharing concerns, and even discussing challenges outside the scope of the nursing curriculum, such as clinical hours, stress management, and balancing responsibilities. Tutors described these relationships as mutually enriching, while tutees reported feeling less isolated and more connected to their academic community. These findings are supported by Koo et al. (2024), who emphasized the social integration and community-building capabilities of peer tutoring in health education. In addition to cognitive and academic growth, the emotional benefits of peer tutoring were prominently featured in the data. Tutees often entered the program with significant hesitancy, expressing feelings of nervousness, fear of judgment, and stigma associated with seeking academic support. However, these feelings were consistently reported to diminish over time as tutees experienced the supportive, empathetic, and non-threatening nature of peer sessions. This process aligns with the emotional trajectory identified by Kang et al. (2021), Ephraim (2020), Mottershead & Ghisoni (2021) and Lockspeiser et al. (2008), who found that well-structured peer environments foster emotional safety, alleviate academic anxiety, and enhance student confidence. Tutors also described the emotional and motivational rewards they experienced from participating in the program. The majority expressed that helping others brought them a deep sense of purpose and satisfaction. Some noted that the experience rekindled their love of learning or affirmed their passion for teaching. Several tutors indicated that tutoring had influenced their career aspirations, with some expressing interest in pursuing roles as clinical instructors or university professors in the future. These insights align with McAtee et al. (2025) and Benè & Bergus (2014), who have emphasized that mentorship roles contribute to the formation of leadership identity, reflective practice, and academic ambition in nursing students. Moreover, tutors cited improved patience, empathy, and adaptability—skills that are crucial in both education and clinical practice.

In this study, tutees reported that peer tutoring effectively clarified complex concepts, particularly in challenging courses such as microbiology, epidemiology, and biostatistics. The majority of students reported improvements in their grades and study habits, transitioning from passive learning methods, such as reading slides, to more active strategies, including discussion, repetition, and the use of multimedia resources. This self-reported academic improvement is consistent with previous research by Al Yahyaei et al. (2024), who found that peer tutoring enhances comprehension and leads to improved exam performance among nursing and health sciences students. Wierzbinski-Cross (2017) explains that participating in peer tutoring helps tutors gain confidence and improve professionally, as they learn to think critically and identify strengths and weaknesses in their own work. This has been demonstrated to be a beneficial supplementary pedagogical approach for enhancing learning efficacy ( Kang et al., 2021). The majority of students consider this practice beneficial, and the authors argue that peer tutoring can enhance students’ motivation to study ( Thomson, Smith & Annesley, 2014). Additionally, this study examines peer tutoring, a partnership system in the learning process, where students assist one another in learning and developing independence through teaching. Peer tutoring not only improves academic skills but also fosters a sense of belonging, reduces students’ feelings of isolation, and promotes collaborative and supportive learning during their studies ( Kim et al., 2021). Additionally, study findings indicated that integrating peer tutoring demonstrates a strong commitment to creating a student-focused learning environment that aligns with global best practices in higher education. Peer tutoring emphasizes student learning over teacher instruction. Peer tutoring has been shown to improve the academic performance of nursing students and decrease their failure rates ( Villanueva, 2023).

Despite the positive experiences, the study identified several areas of barriers or difficulties. Tutors reported challenges in accommodating different learning styles and levels of academic preparedness. Some found it difficult to keep students engaged when they lacked motivation or relied too heavily on the tutor without developing their own study habits. Others faced practical issues such as time constraints, scheduling difficulties during exam periods, and limitations posed by online sessions. These challenges reflect concerns raised by Busby et al. (2021) and Hull (2024), who argue that for peer tutoring programs to succeed, tutors must be trained not only in academic content but also in pedagogical methods, communication strategies, and boundary setting. Another significant barrier identified was the complexity of academic writing and terminology. Both tutors and tutees noted that the structure, vocabulary, and expectations of academic nursing texts often created confusion. Tutees frequently misunderstood assignment requirements or exam questions due to the phrasing rather than the content. Tutors who had faced similar struggles in the past were uniquely positioned to assist their peers in breaking down and interpreting academic language. This reinforces the conclusion of Kim et al. (2021), Hampe et al. (2024), and Sato & Hodge (2009) that peer tutoring can be a critical intervention for language equity in nursing education. Notably, the study also highlighted the transformative potential of peer tutoring in fostering professional development. The majority of participants described how tutoring taught them to be more patient, to adapt to various learning needs, and to view nursing as not only a clinical profession but also one grounded in teaching, advocacy, and mentorship. These competencies are vital in today’s healthcare environments and align with the broader goals of nursing education to produce holistic, adaptable, and collaborative practitioners ( Cupelli & Colalillo, 2025).

The sense of academic partnership and mutual encouragement promoted through these sessions made students feel less alone in their struggles and more capable of succeeding. These findings support the work of Kim et al. (2021), who have emphasized that collaborative learning environments reduce the risk of dropout and improve academic persistence. The findings of this study demonstrate that peer tutoring within the BSN program at the University of Sharjah is a multifaceted and effective educational strategy. It not only supports academic success but also nurtures emotional well-being, social integration, and professional readiness. These outcomes underscore the importance of recognizing peer tutoring as a vital component of nursing education. By offering structural support, training, and ongoing evaluation, universities can enhance the quality and sustainability of peer-led initiatives, ultimately contributing to the development of competent, compassionate, and community-oriented nursing professionals. These benefits were particularly evident in contexts where language posed a barrier to learning. Tutors frequently reported that their tutees, the majority of whom came from Arabic-medium educational backgrounds, struggled not with the core concepts themselves, but with the academic English used in lectures, textbooks, and assessments. Tutors described having to explain or translate medical terminology to make content more accessible—a task that not only helped the tutee but also prompted the tutor to examine and internalize the material from multiple perspectives. These findings align with those of Villanueva (2023), Hampe et al. (2024), and Sato & Hodge (2009), who have highlighted the adverse impact of language barriers on academic confidence and outcomes, particularly among ESL nursing students. By acting as linguistic and academic intermediaries, peer tutors helped bridge this gap, fostering more inclusive and accessible learning environments.

While peer tutoring provides advantages for both general and nursing students, it is essential to acknowledge possible challenges ( Palsson et al., 2017). Some students worry that it may restrict their hands-on clinical experience, lead to conflicts with peers, or result in harmful competition ( Austria, Baraki & Doig, 2013; Stenberg & Carlson, 2015). To ensure peer tutoring is effective, organizing sessions with a clear structure is crucial. This structure should foster a sense of purpose and competence, enhancing students’ knowledge and self-confidence. Psychological empowerment is crucial in this context, enabling students to feel purposeful, capable, and influential in their educational progress ( Bradbury-Jones et al., 2010).

### Implications of the study

The findings of this study have several important implications for nursing education and curriculum development at the University of Sharjah and other institutions with similarly diverse student populations. This research confirms that peer tutoring has a significant impact on the academic performance, emotional resilience, and overall educational satisfaction of nursing students. These outcomes suggest that peer tutoring is not merely a supplementary tool but a vital component of a student-centered, inclusive learning strategy ( Villanueva, 2023). The evidence from this study supports the integration of structured peer tutoring into the core framework of nursing education. Tutees reported enhanced academic understanding, improved exam performance, and the adoption of more effective study strategies. These findings align with those of Kim, Jillapali, and Boyd (2021) and Benè and Bergus (2014), who have demonstrated that peer tutoring contributes to improved exam outcomes and facilitates deeper engagement with clinical and theoretical material. In the context of UOS, where the majority of students come from non-English-speaking backgrounds, the role of peer tutors in breaking down complex material and adapting explanations to suit linguistic and cultural contexts is particularly valuable. Language barriers in healthcare education can significantly affect comprehension and performance ( Sato & Hodge, 2009). Moreover, the emotional benefits observed among participants—such as increased self-confidence, reduced anxiety, and stronger social connection—demonstrate that peer tutoring serves a psychosocial as well as an academic function. These results align with the findings of Kang et al. (2021), Lockspeiser et al. (2008), and Wierzbinski-Cross (2017), who have emphasized that peer learning environments help reduce academic stress by creating collaborative and emotionally safe spaces for learners. At the University of Sharjah, where students often juggle demanding clinical schedules and personal responsibilities, these emotional benefits are critical for maintaining motivation and mental well-being. This study also found that peer tutoring promotes the development of essential professional skills among tutors. The majority of participants expressed a new interest in education, leadership, and mentorship roles, suggesting that peer tutoring could serve as an early platform for cultivating future educators and clinical leaders in nursing. This finding aligns with the observations of McAtee et al. (2025) and Benè & Bergus (2014), who noted that teaching experiences prepare nursing students for broader professional responsibilities. Furthermore, the act of tutoring enhanced students’ abilities in communication, critical thinking, time management, and patience—skills that are indispensable in clinical practice. Another key implication concerns the potential of peer tutoring to address academic isolation. The majority of students shared that participation in the program increased their sense of belonging within the nursing department. This sense of community and connectedness is crucial for student retention and engagement, especially in high-stress academic environments. Our findings support Kim, Jillapali, and Boyd (2021), who found that peer-led support systems contribute to higher retention rates and improved student satisfaction. Given these multidimensional benefits, institutions should take deliberate steps to formalize and support peer tutoring initiatives. This includes developing clear program structures, offering training sessions for peer tutors, and providing recognition or incentives that validate their contributions. Preparing students for mentorship roles through structured training enhances the quality of peer learning and ensures that tutors are equipped to support diverse learners ( Busby et al., 2021).

In summary, the insights gained from this study demonstrate that peer tutoring is a powerful educational intervention that supports academic achievement, emotional health, and professional development among nursing students. For nursing programs seeking to enhance learning outcomes and cultivate a culture of collaboration, peer tutoring offers a flexible, cost-effective, and evidence-based solution. At the University of Sharjah, these findings present a compelling case for integrating peer tutoring into institutional teaching and learning strategies to better meet the evolving needs of nursing students in a competitive and multicultural academic environment.

### Study limitations

The study has several limitations. Since it focuses on a specific group of nursing students from only one university, its generalizability is limited. The findings may not apply to all nursing students, as their demographics and educational backgrounds can vary across different settings or institutions. Additionally, using convenience sampling may introduce selection bias, which can affect the accuracy of the sample’s representation of the broader population. Although reliable tools were employed to assess constructs such as perceived psychological empowerment and peer tutoring effectiveness, reliance on self-reported data may cause response bias. These subjective measures might also overlook some aspects of students’ experiences or perceptions. Finally, while the study identified differences in perceived usefulness between tutors and tutees, it did not explore the specific challenges faced by peer tutors, which could have offered deeper insights into the dynamics of learner interactions.

## Conclusion

At its core, the peer tutoring program was about people helping each other. It wasn’t solely about improving grades; it was about fostering a sense of support and community. Students involved, whether as tutors or tutees, gained more than just academic achievement—they built stronger friendships, rediscovered self-confidence, and gained a better understanding of their personal strengths. Initially, tutees felt nervous, fearing judgment or falling behind, but by the program’s end, the majority had not only enhanced their grades but also gained confidence to ask questions and try new learning strategies. For them, tutoring became more than just studying; it became a vital support system. Tutors, meanwhile, discovered their ability to lead, mentor, and motivate others. Several admitted they never saw themselves as teachers, but through tutoring, they developed newfound confidence. The majority expressed they would gladly participate again, not for credits or pay, but because of the profound meaning of the experience. The strength of peer tutoring is its simplicity: students helping students. It reminds us that learning does not always have to come from a top-down approach. Sometimes, the best assistance comes from someone sitting right next to you, someone who has been through it too and only wants to see you succeed. Moving forward, this program has the potential to grow into something even greater. With more structure, recognition, and integration into university life, peer tutoring could become a vital component of nursing education, a space where knowledge, compassion, and community converge to shape the nurses of tomorrow.

## Data Availability

Participant data contains sensitive personal information, and sharing such data publicly could compromise confidentiality and anonymity. The Institutional Review Board (IRB) has mandated that data sharing is permissible only under specific conditions that ensure participant privacy and align with ethical guidelines. Access to the data may be granted to qualified researchers for legitimate academic purposes upon request. Requests for access must be submitted in writing to the corresponding author, Dr. Richard Mottershead richmottershead@gmail.com.
