# Supplementary material for: Exploring peer tutoring experiences in improving nursing students’ academic success and performance: A qualitative study among Emirati undergraduate nursing students
Source: F1000Res. 2026 Feb 17;14:1416. Originally published 2025 Dec 19. [Version 2] doi: 10.12688/f1000research.172023.2 (PMC12775669; doi:10.12688/f1000research.172023.2)
Supplement: Peer review history

# Reviewer report: Reviewer response for version 2

**DOI:** 10.5256/f1000research.195129.r459307

**Published:** 2026-02-24

**Authors:** Dr. Phillip Quirke

**Affiliations:** 1 Quirke Consultancy Ltd, Tobago, Trinidad and Tobago

No further comments required as all the suggestions and edits required in the initial Review Report have been addressed.

Is the work clearly and accurately presented and does it cite the current literature?

Yes

If applicable, is the statistical analysis and its interpretation appropriate?

Not applicable

Are all the source data underlying the results available to ensure full reproducibility?

Yes

Is the study design appropriate and is the work technically sound?

Yes

Are the conclusions drawn adequately supported by the results?

Yes

Are sufficient details of methods and analysis provided to allow replication by others?

Partly

Reviewer Expertise:

Education Leadership & Management, Teacher Education, Instructional Methodologies, & Reflective Writing.

I confirm that I have read this submission and believe that I have an appropriate level of expertise to confirm that it is of an acceptable scientific standard.

# Reviewer report: Reviewer response for version 2

**DOI:** 10.5256/f1000research.195129.r459308

**Published:** 2026-02-18

**Authors:** Syed Fahad Javaid

**Affiliations:** 1 United Arab Emirates University, Al Ain, United Arab Emirates

Thank you for the opportunity to review this interesting manuscript. I appreciate the authors’ careful consideration of my previous comments and their thorough revisions in response. In light of these changes, I am satisfied that my comments have been adequately addressed and I have no further suggestions to offer.

 I wish the authors every success with their work.

Is the work clearly and accurately presented and does it cite the current literature?

Yes

If applicable, is the statistical analysis and its interpretation appropriate?

Not applicable

Are all the source data underlying the results available to ensure full reproducibility?

Partly

Is the study design appropriate and is the work technically sound?

Yes

Are the conclusions drawn adequately supported by the results?

Yes

Are sufficient details of methods and analysis provided to allow replication by others?

Partly

Reviewer Expertise:

Medical education, Mental Health, Socioeconomic barriers to healthcare access

I confirm that I have read this submission and believe that I have an appropriate level of expertise to confirm that it is of an acceptable scientific standard.

# Response

**Published:** 2026-02-18

**Authors:** Dr. Richard Mottershead

**Affiliations:** College of Health Sciences, University of Sharjah, Sharjah, Sharjah, United Arab Emirates

On behalf on the research team, I thank esteemed Prof. Javaid for his thoughtful comments and we believe our article has been enhanced through his contributions. 

 kindest regards 

 Dr. Richard Mottershead

# Reviewer report: Reviewer response for version 1

**DOI:** 10.5256/f1000research.189702.r443640

**Published:** 2026-01-06

**Authors:** Dr. Phillip Quirke

**Affiliations:** 1 Quirke Consultancy Ltd, Tobago, Trinidad and Tobago

This article provides a description of a peer tutoring experience with a small group of 20 multi-lingual, multi-cultural students in the University of Sharjah, UAE. It uses appropriate qualitative methodologies (Semi-structured interviews, COREQ and Inductive Content Analysis), and the resulting themes are well described although the Results section needs editing and review. The conclusion is well articulated and the final sentences provide an impactful closing.

 The article meets a gap in current literature on peer tutoring in multi-cultural and multi-lingual student populations in the UAE.

 There are two areas where further detail on methodology would have enhanced the article: First, on page five in the Research Instrument section, detail on how the "extensive literature review" had been conducted with references cited would have been useful for readers; and secondly, on page six at the end of the first sentence in the Data Analysis section, "following recommended procedures" requires either a reference or possibly the 'Elo & Kyngas' reference could be moved here to the end of the sentence.

 The analysis and interpretation of the qualitative data is appropriate. 

 The source data underlying the results is available upon contacting the correspondent author if required.

 The conclusions drawn are adequately supported by the results although the Results section needs to be edited as follows:

 In the introductory paragraph of the Results section, the authors state:

 "Each main theme has sub-themes. Tutors reported five themes, and tutees identified three themes. Peer tutoring barriers have four sub-themes." But in the following theme descriptions, there are SIX sub-themes reported by the Tutors and THREE sub-themes reported for the Barriers. 

 Only the Tutee sub-themes are numbered.

 So, the Tutor and Barrier sub-themes should be numbered, and the Final Tutor Sub-theme should be moved to the Barriers.

 The introductory paragraph of the Results section would then read correctly and the whole section would be more coherent and cohesive

 .

 I would also suggest that the red font for tutor and tutee quotes is removed, and quotes left in italics.

 There are also two format issues with in-text citations.

 1 - On page 4, penultimate paragraph: "where feedback is viewed as an opportunity for growth rather than criticism ( Muthalib et al., 2023) and Mottershead & Alonaizi (2022)" should read:

 "where feedback is viewed as an opportunity for growth rather than criticism ( Muthalib et al., 2023; Mottershead & Alonaizi, 2022)."

 2 - On page 5 in the Design paragraph: "By adopting a qualitative research design, researchers can gain a deeper understanding of the complex and comprehensive nature of the studied topic ( Creswell, 2012) and Mottershead et al. (2024)" should read:

 "By adopting a qualitative research design, researchers can gain a deeper understanding of the complex and comprehensive nature of the studied topic ( Creswell, 2012; Mottershead et al., 2024)."

 Finally there are two required text edits:

 1 - On p. 8, the second quote under Communication Skills reads " *(Tutors 4)" *and this should be: " *(Tutor 4)"*

 2 - On p. 11, the penultimate sentence reads: "Additionally, this study examines peer tutoring, a partnership system in the learning process, where students assist one another in learning and developing independence through teaching.", but this is NOT additional, so the sentence should begin:

 "This study examines ..."

Is the work clearly and accurately presented and does it cite the current literature?

Yes

If applicable, is the statistical analysis and its interpretation appropriate?

Not applicable

Are all the source data underlying the results available to ensure full reproducibility?

Yes

Is the study design appropriate and is the work technically sound?

Yes

Are the conclusions drawn adequately supported by the results?

Yes

Are sufficient details of methods and analysis provided to allow replication by others?

Partly

Reviewer Expertise:

Education Leadership & Management, Teacher Education, Instructional Methodologies, & Reflective Writing.

I confirm that I have read this submission and believe that I have an appropriate level of expertise to confirm that it is of an acceptable scientific standard.

# Response

**Published:** 2026-02-18

**Authors:** Dr. Richard Mottershead

**Affiliations:** College of Health Sciences, University of Sharjah, Sharjah, Sharjah, United Arab Emirates

On behalf of the research team, I would like to thank the esteemed reviewer Dr. Phil Quirke for his very positive feedback and we will be sure to take on board his suggestions for future articles. 

 Again, thank you for your time and consideration of our research. 

 Dr. Richard Mottershead

# Reviewer report: Reviewer response for version 1

**DOI:** 10.5256/f1000research.189702.r443645

**Published:** 2026-01-05

**Authors:** Syed Fahad Javaid

**Affiliations:** 1 United Arab Emirates University, Al Ain, United Arab Emirates

Thank you very much for the opportunity to review this manuscript. The authors have explored an interesting area of research. This manuscript presents a qualitative study looking into the relationship between peer-tutoring and academic performance among undergraduate nursing students in the United Arab Emirates. It is an important topic with local relevance.

 The manuscript is clearly written. Themes are clearly delineated and flow logically from the data.

 The title is reasonable and clearly indicates the article type. The abstract is structured and informative, providing a concise snapshot of the study.  However, I recommend that the authors operationalise key terms such as ‘academic success’, like GPA, exam performance, or self-perception, so readers can assess outcomes clearly.

 The introduction provides a sound theoretical foundation and a decent literature review. That said, please strengthen the gap statement by specifying what prior research lacks and how this study fills that gap. 

 The sampling strategy is insufficiently justified; the rationale for including 17 participants and the balance between tutors and students is unclear. Researcher positionality and potential bias are not discussed, and no steps for piloting or validating interview questions are mentioned. Including more on how interviews were conducted and themes were derived would significantly improve reproducibility and transparency.

 The discussion links results to existing literature but tends to overstate the novelty and impact of the study. Please add more recently published UAE or regional studies.

  Limitations are acknowledged but not explored in depth, and there is minimal reflection on potential selection bias or generalisability of results. The conclusion reiterates findings rather than offering new insights or implications. More critical reflection is needed to temper claims and increase scholarly impact.

 Overall, this is a promising and relevant study that contributes to nursing education literature. The authors need to strengthen methodological transparency, clarifying analytical distinctions, and better situating the findings within broader scholarly work.

 Addressing the above points will significantly enhance the academic value of this interesting piece of research.

Is the work clearly and accurately presented and does it cite the current literature?

Yes

If applicable, is the statistical analysis and its interpretation appropriate?

Not applicable

Are all the source data underlying the results available to ensure full reproducibility?

Partly

Is the study design appropriate and is the work technically sound?

Yes

Are the conclusions drawn adequately supported by the results?

Yes

Are sufficient details of methods and analysis provided to allow replication by others?

Partly

Reviewer Expertise:

Medical education, Mental Health, Socioeconomic barriers to healthcare access

I confirm that I have read this submission and believe that I have an appropriate level of expertise to confirm that it is of an acceptable scientific standard, however I have significant reservations, as outlined above.

# Reviewer report: Reviewer response for version 1

**DOI:** 10.5256/f1000research.189702.r443647

**Published:** 2025-12-31

**Authors:** Rebecca George

**Affiliations:** 1 Fatima College of Health Sciences, Ajman, Ajman, United Arab Emirates

This qualitative study explores the experiences of peer tutors and tutees within an undergraduate nursing program at the University of Sharjah, UAE. Using semi-structured interviews and inductive qualitative content analysis guided by COREQ, the authors examine how peer tutoring influences academic performance, emotional resilience, social connectedness, and professional development among Emirati nursing students. The findings identify three overarching themes: tutors’ experiences, tutees’ experiences, and barriers to peer tutoring, highlighting both academic and psychosocial benefits alongside linguistic, time, and engagement challenges.

 Overall, the topic is relevant, timely, and contextually important for nursing education in culturally and linguistically diverse settings. The manuscript contributes valuable qualitative insights, particularly from the Middle Eastern context.

 To further strengthen the methodological rigor and transparency of the study, it is recommended that the authors clearly clarify whether data coding was conducted independently by more than one researcher, explicitly describe the strategies used to enhance trustworthiness and include a brief reflexivity statement outlining the researchers’ roles, positionality, and potential influence on data interpretation.

Is the work clearly and accurately presented and does it cite the current literature?

Yes

If applicable, is the statistical analysis and its interpretation appropriate?

Not applicable

Are all the source data underlying the results available to ensure full reproducibility?

Yes

Is the study design appropriate and is the work technically sound?

Yes

Are the conclusions drawn adequately supported by the results?

Yes

Are sufficient details of methods and analysis provided to allow replication by others?

Partly

Reviewer Expertise:

Nursing Education and Health Professions Education; Critical care nursing

I confirm that I have read this submission and believe that I have an appropriate level of expertise to confirm that it is of an acceptable scientific standard.

# Response

**Published:** 2026-01-07

**Authors:** Muhammad Arsyad Subu

**Affiliations:** University of Sharjah, United Arab Emirates

Dear reviewer,

 Greetings.

 Thank you for reviewing our paper and for your positive comments. It is greatly appreciated. 

 Best regards,
